# Depression, cognitive impairment and dementia: Why should clinicians care about the web of causation?

**Published:** 2009-01

**Authors:** Mary Ganguli

**Affiliations:** Professor of Psychiatry, Neurology, and Epidemiology, University of Pittsburgh School of Medicine and Graduate School of Public Health, Pittsburgh, PA 15208, USA

**Keywords:** Aging, Alzheimer’s disease, cognitive decline

## Abstract

Depression, cognitive impairment and dementia are all common in older adults. The relationship between them is bi-directional and complex. The literature on the subject is growing and fascinating but also riddled with apparent inconsistencies. This brief review attempts to clarify and integrate information from clinical, laboratory, and community studies and to draw some inferences of potential relevance to clinicians.

## RELEVANCE AND SCOPE

As standards of living improve across the globe, people are living longer, and the world’s population is aging rapidly.[1] Although the proportions of people aged 60 or more years are smaller in the “developing” countries than in the “developed” countries, the sheer numbers of older adults will by 2020 be much larger in the low and middle-income countries of the world than in the affluent countries. Thus, depression and dementia will soon become major public health problems in countries such as India.[2,3] Depression and dementia are both common in older adults; cognitive functioning declines slightly with normal aging; depression itself can be associated with cognitive impairment and dementia. Increasingly, there are intriguing reports that suggest depression might itself increase the risk of dementia.[4] This article will briefly and selectively review the evidence and draw some broad inferences about the relationships among these conditions. This review is not intended to be comprehensive or systematic, and will not touch on several important, interesting, and relevant issues such as genetics, pharmacology, lifestyle factors, neurochemistry, biomarkers, or neuroimaging.

There are both broad similarities and wide discrepancies among different studies of depression, dementia, and cognitive impairment and decline. A key issue in understanding the similarities and discrepancies is that different studies use different measurements and definitions, and examine very different groups of people. In the clinical psychiatry setting, we might see something very different in our own patients to what is seen in neurology or general practice, or what is reported in the literature from a population-based epidemiologic study. Cross-sectional and longitudinal studies may appear to show different relationships. Observational and interventional studies may appear to have conflicting results. To avoid becoming cynical and frustrated ourselves by all the apparent inconsistency, it is helpful to gain a more integrated understanding of the literature and its implications for clinicians. A useful epidemiologic concept is the “web of causation,” meaning - in this case - that most mental disorders do not have a single cause but are rather the result of multiple factors that interact with one another in different ways at different points along the life course.

## DEFINITIONS

First, we must define what we mean by depression. Psychiatrists usually mean a depressive illness, characterized by depressed or otherwise low mood, sustained over several days. This mood change itself occurs in the presence of a few other features, such as disturbed sleep and/or appetite, energy, interest, low self-regard, perhaps guilt, pessimism or hopelessness, and thoughts of death. Depending on the diagnostic classification system being used, a depressive episode in ICD-10[5] or a major depression in DSM-IV-TR[6] requires a certain number of these symptoms to be present over a certain period of time, usually two weeks. However, non-psychiatric practitioners may not follow these definitions as strictly as we do. Patients and their relatives may speak of being depressed without consulting all these books and criteria, while some who fulfill the criteria may not call themselves depressed. Further, obtaining a history of a previous depressive episode may be difficult because precise details are not always easy to remember. In many studies, cognitive impairment and dementia have been examined in relation to well-defined episodes of depressive illness established by expert clinical assessment of patients presenting for treatment.[7–11] However, in many other studies, especially of community-based samples, depression has been measured by scores on symptom rating scales.[12,13]

Next, we should look at what we mean by cognitive impairment. In most studies, the term refers to poor performance on one or more standardized tests of cognitive functioning. Sometimes these are general mental status tests, and sometimes they are large, comprehensive batteries of tests of different cognitive functions. We know of course that cognitive test performance is heavily influenced by age and education, and, in some cases, by gender.[14] In clinical practice, we find patients or their families talking about cognitive impairment as reflected in impaired functioning, forgetfulness, repetitiveness, difficulty in carrying out tasks that would have been routine in the past, getting lost in familiar places or becoming disoriented. In some patients who report no difficulties, we find deficits on objective testing, and in others with significant complaints, we find no objective deficits. Some clinicians, and some studies, examine subjective reports by individuals or their families, asking standardized questions; others rely solely on objective measures. The term mild cognitive impairment (MCI) has been in use since the early 1990s but about a decade ago it acquired the more specific meaning of a “transitional” or intermediate state between normal cognition and dementia. The precise operational definition of this term has varied across studies and over time[15] with some authors claiming that it is simply early Alzheimer’s disease and not a separate entity.[16]

Cognitive decline is more difficult to pin down. A certain amount of change, particularly in the speed of information processing, is seen with normal aging.[17] However, if given the same test year after year, we become practiced at it and may show no change or even an initial improvement in performance; this learning effect may conceal the mild loss of ability which is occurring in the background.[18] It can be quite challenging to differentiate between the minimal cognitive changes of normal aging and those of early dementia. Depression also has a negative effect on processing speed, in a way exaggerating the effect of normal aging.[8]

Fortunately, most health care providers have the same understanding of the term dementia; most of us today do not use the term “senility” or consider dementia to be simply a sign of old age. However, there is an unfortunate tendency in much of the literature for authors to use the words “dementia” and “Alzheimer’s disease” as though they mean the same thing. On the other hand, sometimes patients or families ask, “Do I have dementia or do I have Alzheimer’s disease?” According to current definitions, dementia refers to a syndrome of acquired cognitive impairment, occurring in clear consciousness, sufficient to interfere with social and occupational functioning, and characterized by impairment in at least two cognitive domains.[6] For the past few decades, the definition has required that memory be one of the domains that is impaired, but this requirement may not persist in future revisions of diagnostic criteria. For example, it is possible that, in the future, an individual with significant impairments in executive functioning and language functioning can be diagnosed as having dementia, even if memory has not been affected.

Alzheimer’s disease is a primary neurodegenerative disease which appears to be the single most common cause of the dementia syndrome in older adults in most populations which have been studied around the world.[3] The definitive diagnosis of Alzheimer’s disease is made post-mortem on the basis of classic neuropathology: cortical atrophy, amyloid plaques, and neurofibrillary tangles, seen particularly in the temporoparietal regions. Ante-mortem diagnosis is made clinically[19,20] and many experts now believe that hippocampal atrophy can be seen on magnetic resonance imaging (MRI) brain scans very early in the disease.[21–23] The high proportion of dementias caused by Alzheimer’s disease has contributed to the terms “dementia” and “Alzheimer’s” being used interchangeably. It has also led to the diagnostic criteria for the dementia syndrome being “Alzheimerized” by the requirement of specific impairment in memory.[24]

The next single most frequent cause of dementia is cerebrovascular disease; either cortical infarcts or white matter disease, or both, can lead to significant cognitive impairment and decline. However, particularly among the very old, it is more common to see Alzheimer’s and vascular pathology co-existing than to see pure vascular disease with no concomitant degenerative disease. To complicate matters further, many vascular factors increase risk of Alzheimer’s disease.[25] Cerebrovascular disease, whether or not an overt stroke has occurred, is also well-established as a source of depression.[26,27] Other less frequent primary brain disorders causing dementia include frontotemporal dementia, dementia with Lewy bodies, Parkinson’s disease, progressive supranuclear palsy, etc. Each of these has distinctive clinical and pathological features and many of them have behavioral manifestations including depression; however, these conditions, and non-CNS conditions that cause or contribute to dementia, are beyond the scope of the current review.

The concept of “brain reserve” was introduced by Katzman[28] in the context of an autopsy series in which almost all the brains examined had evidence of Alzheimer-type pathology, but not all of the individuals had manifested dementia during life. It appeared that those who did not dement clinically, despite pathology being present, were those who had larger brain mass and better preserved large neurons. Katzman theorized that the brains of these individuals had a reserve capacity that allowed them to compensate for the pathology sufficiently that they could continue to function within the normal range. Another autopsy study showed that individuals who remained non-demented despite degenerative neuropathology were those who did not have concomitant subcortical cerebrovascular disease which presumably disrupted circuits and further compromised their brain reserve.[29] A parallel concept is that of “cognitive reserve” which may derive partly from brain reserve and partly from other factors that increase cognitive capacity, such as premorbid intelligence, education, and mental stimulation.[30] While neither brain reserve nor cognitive reserve prevent the development of pathology, they might help to delay or mitigate the clinical symptoms of dementia. It must be clearly emphasized that the above are only conceptual models.

## RELATIONSHIPS OF DEPRESSION WITH COGNITIVE IMPAIRMENT, DECLINE, AND DEMENTIA

**Depression and cognitive impairment:** A profusion of studies have demonstrated that the presence of depression is associated with worse performance on cognitive tests, both in clinical samples of patients with depressive illnesses and in population-based samples of older adults drawn from the community.[4–13] Many older patients with depression complain of difficulty in concentrating and remembering, and this subjective phenomenon is borne out by objective studies showing that cognitive deficits in depression are mediated almost entirely by slowed processing speed and working memory (executive functioning).[7–9] There is some variation in results as to which cognitive domains are associated with depression, related in part to the nature of the study population.

**Depression and cognitive decline:** There is less agreement in the literature about the relationship between depression and continued (progressive) cognitive decline over time. Some treatment trials of patients with late-life depression have shown that, although cognitive functioning improves as depression remits, there is a residual cognitive deficit that remains present.[10,11,31] One community-based prospective study found that depression was associated with increased risk of subsequent MCI.[13] However, another similar study, which categorized participants into those who went on to develop dementia and those who remained dementia-free, found that the former group declined substantially while the latter group did not. Depression at baseline did not predict cognitive decline in either group. In other words, substantial cognitive decline was a function of underlying/incipient dementing illness rather than of depression.[12]

**Depression and dementia:** Many patients with Alzheimer’s and other dementias have depressed mood and other behavioral symptoms.[32,33] Some studies have shown that these patients report depression well before they report cognitive difficulties, raising the possibility that depression was a risk factor for dementia. Meta-analyses of the world literature suggested that history of depression was a risk factor for dementia[34] and Alzheimer’s disease in particular.[35] To quote a recent, comprehensive review, “the temporal association between cognitive and depressive symptoms in elderly patients varies widely, yet increasing evidence suggests that depressive illness contributes to the development of persistent or progressive cognitive deficits in some patients”.[36] One population-based study that examined the temporal sequence concluded that although depressive symptoms preceded the onset of dementia symptoms, depression appeared to be a prodrome rather than an independent predictor of dementia.[37] Thus, depression might be a prodromal or early sign of dementia rather than a separate condition or risk factor for dementia.

**Depressive pseudodementia vs. pre-dementia:** It had been observed by clinicians for decades that when undergoing cognitive testing, depressed patients would make minimal effort; they would often lose points not by giving wrong answers but by responding, “I don’t know.” It was believed that, in contrast, patients with dementia were more likely to give wrong answers, and therefore that “don’t know” responses might provide a clue as to the true diagnosis. When these patients recovered from their depressions, they would no longer respond in this way; they would perform better on tests and appear restored to their previous level of cognitive functioning. The term “depressive pseudo-dementia” was introduced to describe this phenomenon, implying that the temporary dementia-like syndrome was in fact due entirely to depression. This was a comforting concept that encouraged us to look harder for evidence of depression in cognitively impaired patients, and to treat a lot of depression that might otherwise have been left untreated. However, objective research has shown that patients with dementia are as or more likely than depressed patients to say “I don’t know”.[38] Further, as depressed, cognitively impaired patients were systematically studied and followed over time, a significant proportion continued to experience progressive cognitive decline even though they were no longer depressed.[39] Thus, it appeared that the impairment seen during the depression was not “pseudo” but a pre-dementia harbinger of more permanent cognitive decline in the future. As disappointing as this was to clinicians, it opened the door to new thinking about the relationship between depression and dementia.

## POSSIBLE MECHANISMS

Depression could be a psychological response to the individual’s self-awareness of mild cognitive deficits that have not yet begun to interfere with daily functioning. Individuals experiencing the earliest signs of incipient dementia may realize that something has changed and is not quite right, without necessarily being able to say what exactly is wrong. Routine tasks become difficult and effortful, and previously enjoyed activities (e.g., reading, card games) are no longer pleasurable. A natural response might be to withdraw from these activities, leading others to assume that the person is suffering primarily from depression and anhedonia. Individuals with more advanced deficits might realize what is wrong; most people would become sad at the prospect of having an incurable progressive disease leading to loss of autonomy in even the most basic functions. Other individuals whose dementia manifests in part as anosognosia, or loss of insight, might not react with depressed mood.

Depressive symptoms might be manifestations of the same underlying disease that has caused the dementia, whether Alzheimer’s or Parkinson’s diseases or another condition.[40,41] Along with cholinergic system disruptions causing memory loss, it is entirely possible that the progressive brain disease affects serotonergic and noradrenergic systems as well. In addition, some dementia patients exhibit emotional liability with tearfulness, which is often mistaken for depression. Post-stroke depression is a well-known phenomenon which often does not respond well to standard antidepressant treatment.[26] A broader phenomenon is “vascular depression”[42,43] denoting the presence of cerebrovascular disease but not necessarily an overt stroke. It is hypothesized to operate by disruption of prefrontal systems that mediate both mood and executive functions.

When individuals with progressive brain disease finally manifest a clinical dementia syndrome, subclinical pathology has likely been developing for at least a few years before the onset of symptoms. Symptoms appear when the individual can no longer compensate functionally for the underlying pathology. Symptom onset can be viewed as a tipping of the balance between the amount of pathology in the brain and the individual’s ability to compensate. Either the amount of pathology has increased till it crossed the clinical threshold, holding compensatory ability constant, or the compensatory mechanisms have become compromised to the extent that deficits become apparent. This ability to compensate can be conceptualized as part of the cognitive reserve discussed earlier.[30] Depression may compromise cognitive reserve and allow symptoms of dementia to be manifested earlier than they would have been otherwise. This could create a false impression that the cognitive impairment is “caused” (rather than unmasked) by the depression. If the depression remits, compensatory ability could be restored and the patient may appear unimpaired for a while.

Some unfortunate individuals may have two separate illnesses, a primary depressive disorder as well a dementing disorder; this conclusion is usually drawn when the individual has suffered recurrent depressive episodes earlier in life, and now has a dementia as well. A question that has emerged over the past several years is whether major depression in earlier life is an independent risk factor for Alzheimer’s disease in later life. Originally, case-control studies in memory disorders clinics showed that patients with Alzheimer’s were significantly more likely than healthy control subjects to have had a previous history of major depression.[44,45] Similar findings were reported from population-based studies.[46,47] Depression, like stress generally, raises cortisol levels.[48] Animal studies have demonstrated that prolonged hypercortisolemia leads to hippocampal atrophy in rats.[49,50] Hypothalamic-pituitary-adrenal axis dysfunction in depression and Alzheimer’s disease has distinct MRI correlates.[51] Neuroimaging studies have shown that depressed older adults have hippocampal atrophy proportional to the duration of untreated depression.[52] In animal studies, stress-induced neuronal atrophy in the hippocampus, as well as spatial memory deficits, are actually reversible.[53] In an autopsy study of people who died with Alzheimer’s disease, those with a previous history of major depression had greater density of amyloid plaques and neurofibrillary tangles in the hippocampus than other people who died with Alzheimer’s but did not previously have depression.[54]

This theory suggests that depressive illness, particularly if prolonged and inadequately treated, may result in sustained elevations of serum cortisol. Hypercortisolemia in turn may lead to hippocampal damage, reducing its ability to resist or compensate for degenerative damage from Alzheimer’s disease. Imagine two individuals with incipient Alzheimer’s disease pathology in their brains. One of them has also suffered with depression over the years while the other did not. Both will develop clinical symptoms of dementia, if they live long enough, but the former will develop symptoms earlier than the latter, because the former’s hippocampus was already damaged by the depression. Depression did not cause the dementia, but increased the individual’s susceptibility to the dementia caused by Alzheimer’s disease. This theory holds out the hope that early, effective detection and treatment of depression could not only relieve depression but also prevent or delay future dementia. However, long-term human studies have not yet tested this hypothesis.

## CONCLUSIONS

Thus, there are several ways in which depression could be related to dementia and cognitive impairment. First, both being common conditions, they could occur together in the same individual by chance alone. Second, in some individuals, cognitive impairment and depressive symptoms could both be manifestations of the same brain disease. Third, individuals experiencing cognitive deficits could become depressed as a reaction to recognizing their losses and poor prognosis. Fourth, depression might unmask a dementia which had until then remained undetected. Fifth, depression itself could be an independent risk factor for the future development of dementia; this seems a more plausible explanation when the individual has had early-onset recurrent or chronic depression than if the depression occurs for the first time shortly before the dementia is manifested (Note that the term “risk factor” does not imply that depression is the cause of dementia, but only that it increases the probability of dementia). Finally, these are not mutually exclusive possibilities.

What does all this mean for clinicians? Clearly, it is important that we actively monitor our older patients for both depression and dementia. A useful clinical adage is “when you see depression, suspect dementia; when you see dementia, suspect depression.” We should counsel all patients to minimize cardiovascular risk factors and maintain good “heart health” because what is good for the heart is usually good for the brain as well. In the same way that we do not dismiss patients’ depressive symptoms, we should also avoid brushing off their complaints about cognitive impairment. Unverifiable reassurances such as “you don’t have Alzheimer’s,” or “it is just old age” should be avoided. We should instead take patients’ concerns seriously, obtain a good history, test them objectively, and investigate potential modifiable causes and contributory factors. If the investigations are negative, we can explain that we have found no current evidence of anything serious and promise to monitor their cognition as time goes on. However, we should use the opportunity to treat any treatable conditions that are found, including but not limited to depression, without making promises or predictions about relief of cognitive symptoms.

It is always clinically appropriate to treat depression to reduce suffering caused by depression, which itself is a major cause of morbidity and mortality.[55] Perhaps in a few years we will know whether treating depression, as an added bonus, will also stave off dementia.
